# Systematic review of prevalence, risk factors, and management of instability following reverse shoulder arthroplasty

**DOI:** 10.1016/j.xrrt.2022.02.009

**Published:** 2022-03-30

**Authors:** Jeffrey J. Olson, Michael D. Galetta, Rachel E. Keller, Luke S. Oh, Evan A. O'Donnell

**Affiliations:** a Harvard Combined Orthopaedic Residency Program, Boston, MA, USA; b Department of Orthopaedic Surgery, Massachusetts General Hospital, Sports Medicine Service, Boston, MA, USA; c Department of Orthopedic Surgery, Philadelphia College of Osteopathic Medicine, Philadelphia, PA, USA

**Keywords:** Reverse shoulder arthroplasty, Instability, Systematic review, Risk factors, Prevalence, Management

## Abstract

**Background:**

Since its approval for use, reverse shoulder arthroplasty (RSA) has become the primary treatment for cuff tear arthropathy, with indications expanding more recently to include revision fracture, osteoarthritis with significant glenoid bone loss, tumor, and chronic instability. Instability is a well-described postoperative complication, occurring in 1to 31% of relatively small cohorts and case series. Given the relative infrequency of instability, there remains a need for a comprehensive review of instability with a focus on risk factors and management. Our goal of this systematic review is to describe the prevalence, risk factors, and management strategies for instability following RSA.

**Methods:**

A systematic review of the PubMed, EMBASE, MEDLINE, Scopus, and Cochrane Library databases was performed according to PRISMA guidelines. Inclusion criteria included primary RSA cohorts ≥ 100 patients, revision RSA cohorts of any size, and minimum 1-year follow-up. The primary outcome of interest was postoperative instability. MINORS criteria were used to assess study bias. Descriptive statistical analysis was performed with data reported as ranges.

**Results:**

Seventeen studies that included 7885 cases of RSA were reviewed. The mean follow-up ranged from 12 to 84 months. Mean age ranged from 64 to 77 years old, and males represented 19 to 39% of cohorts. There were 204 (2.5%) dislocations in 7885 cases, accounting for a rate of instability from 0.4 to 49% across all studies. By intervention, instability rates ranged from 1 to 5% (primary RSA cases), 1 to 49% (revision RSA cases only), and 0.4 to 10% (mixed cohorts). Subscapularis insufficiency and proximal humerus fractures, and fracture sequelae (malunion and nonunion) were identified as risk factors for instability. Closed reduction and casting and revision RSA were reported as successful treatment strategies with acceptable rates of stable prostheses (28-100% and 55-100%, respectively, across studies). Hemiarthroplasty or resection arthroplasty due to recurrent instability was not uncommon after 2 or more episodes of instability.

**Conclusion:**

Instability following RSA occurs infrequently (1-5%) following primary RSA and more commonly following revision RSA (1-49%). RSA for acute proximal humerus fracture and fracture sequelae carries a higher risk of instability. Subscapularis repair appears to be a protective factor. While instability may be successfully treated with closed management or revision RSA, recurrent instability may ultimately require hemiarthroplasty or resection arthroplasty.

Since its introduction by Paul Grammont in the 1980s and approval for use in the United States in 2004, reverse shoulder arthroplasty (RSA) has become a popular and effective treatment for myriad shoulder conditions.^12^^,^^18^^,^^19^^,^^33^^,^^36^ First indicated for cuff tear arthropathy, the indications for RSA have rapidly expanded to the treatment of glenohumeral osteoarthritis with significant deformity, massive irreparable rotator cuff tears, proximal humerus fractures acutely, as well as their sequelae, tumor-resections, and revision surgery;^5^^,^^10^^,^^19^^,^^36^ as a result, the utilization of RSAs continues to rise within the United States.^5^ While preliminary long-term studies have shown efficacy and durability,^6^ complication rates, including prosthetic instability (1-31%) remain a concern.^5^^,^^6^^,^^33^^,^^34^

While instability is well described, there remains a need for a comprehensive review of prevalence rates, risk factors, and the management of this challenging complication. Current estimations of the prevalence of instability after RSA are limited by small cohort studies and varying indications.^8^^,^^11^^,^^13^^,^^26^^,^^27^^,^^36^^,^^37^ While recent systematic reviews report pooled prevalence rates, they fail to provide an in-depth review of risk factors and the management of instability.^1^^,^^20^^,^^28^ Thus, the goals of this study were three-fold: (1) report the prevalence of instability following RSA (2) identify risk factors associated with increased rates of instability and (3) to evaluate the management of instability based on previous studies results.

## Materials and methods

### Search strategy

This systematic review was performed in accordance with Preferred Reporting Items for Systematic Reviews and Meta-Analyses (PRISMA) guidelines and registered on PROSPERO (ID: 200992). In August 2020, PubMed, EMBASE (Elsevier), MEDLINE (Ovid), Scopus (Elsevier), and Cochrane Library (Wiley) databases were queried for articles using the explicit search terms 'reverse shoulder arthroplasty, reverse total shoulder arthroplasty, instability, stability, unstable, subluxation, dislocate, and dislocation'.

### Study selection

**Figure 1:**
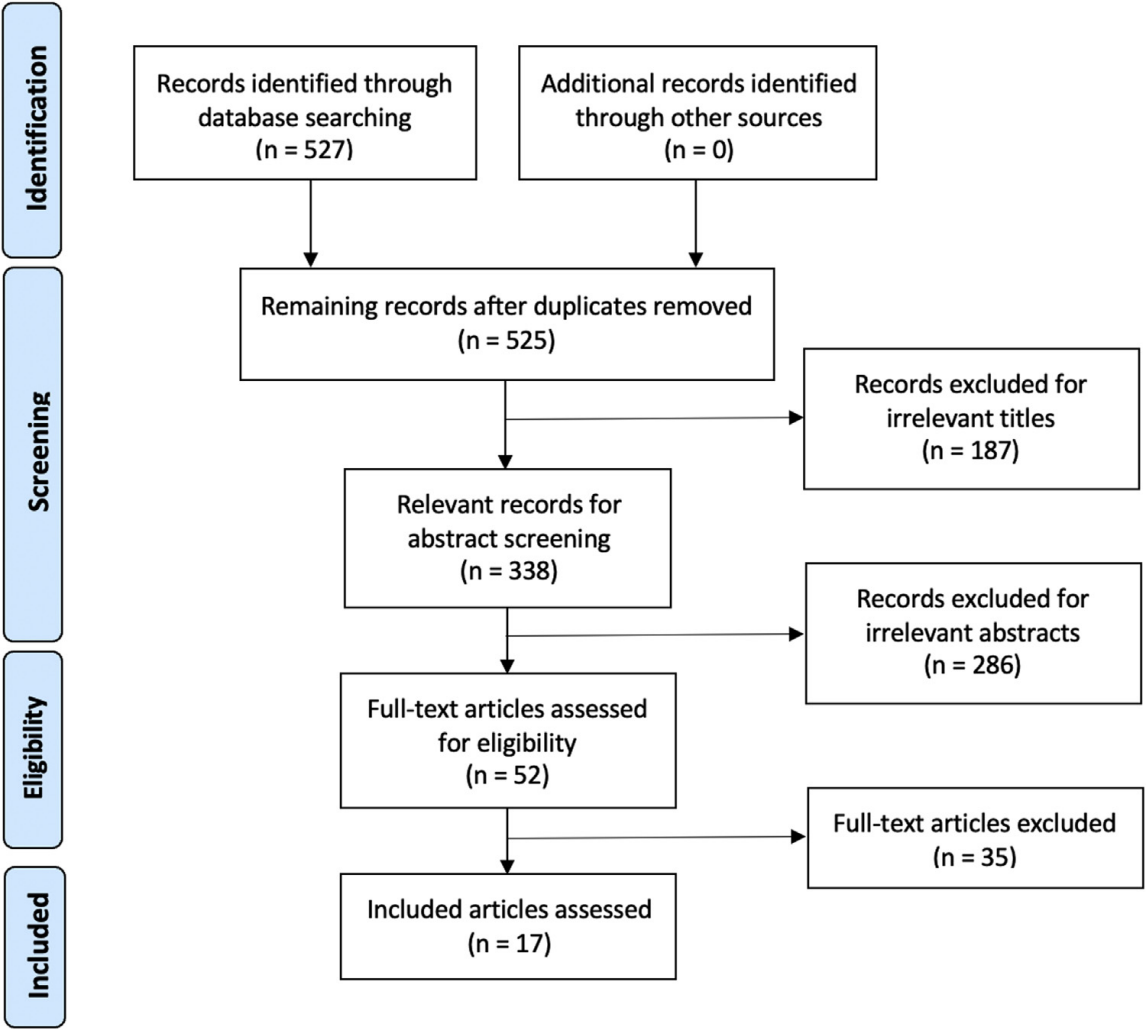
Prisma Flow Diagram.

The screening process was executed in duplicate by two independent reviewers (JJO, MDG) in three stages: title screening, abstract screening, and finally, a full-text review (Fig. 1). Screening was conducted according to strict inclusion/exclusion criteria. Inclusion criteria comprised articles of level IV evidence or higher, primary RSA cohorts ≥ 100 patients, revision RSA cohorts of any size, and minimum 1-year follow-up. The primary indication for RSA of interest was rotator cuff tear arthropathy (CTA), although cohorts with heterogenous indications for surgery were included. Exclusion criteria included review articles, duplicate articles, non-English articles, biomechanical studies, and insurance/administrative database studies, which lacked a granular level of detail sufficient for analysis. Discordant reviews were reconciled by a third independent reviewer (EAO) who made the final decision regarding inclusion or exclusion in the study

### Data extraction

Data of interest extracted from each article included study design, level of evidence (LOE), patient demographics (sex, mean age), minimum and average follow-up, surgical indications, primary vs. revision cohort, number of total RSA's, status of subscapularis (repaired vs. insufficiency or elected nonrepair), number of postop instability complications, the reason for instability, management of instability cases, and prosthesis used. Instability was defined as frank dislocation of the prosthetic glenohumeral joint requiring closed or open reduction.

### Bias and quality assessment

The MINORS criteria were used to assess potential bias in the collected articles.^30^ These criteria score comparative studies on a scale of 0-24 and noncomparative studies on a scale from 0 to 16—the highest value representing the least risk of bias in both scenarios.

### Statistical analysis

Categorical data were reported as frequency (percentage), and continuous data were reported as mean or median (standard deviation or range). Statistical analysis was performed using Microsoft Excel (Microsoft Corporation, 2018. *Microsoft Excel*).

## Results

### Level and quality of evidence

After the preliminary search, a total of 527 articles were collected. After deduplication and screening, 17 articles were included for data extraction (Fig. 1). Out of the 17 studies included, there was no level I evidence, one article was level II, nine were level 3 evidence, and seven were level 4 evidence. According to the MINORS criteria, the average score for comparative studies (n = 8) was 7.4/16, and 11.5/24 for noncomparative studies (9).

### Indications

There was a total of 7885 reverse shoulder arthroplasties in the 17 studies reviewed; 281 (3.5%) were revision arthroplasty cases. The mean follow-up ranged from 12 to 84 months with a minimum of 12 months follow-up. The mean age ranged from 64 to 77 years in the 12 studies reporting age, and the overall proportion of male patients ranged from 19 to 39% in 4552 patients from 9 studies reporting on sex. The most common indications for RSA reported in eight studies (n = 4590 cases) were cuff tear arthropathy (30-79%), massive irreparable cuff tear (7-17%), acute proximal humerus fracture (1-25%), osteoarthritis (6-17%), inflammatory arthritis (1-8%), and revision surgery (0-24%) (Table I). Other less common reasons included tumor and fracture sequelae (i.e., nonunion, malunion).

**Table I:** Demographic and outcome data of individual studies by implant design.

Author & year	Design	N	Sex (M:F)	Mean age	Diagnosis∗	Instability no. (%)	Mean follow-up (Mo)
*Implant*							
**Tornier Aequalis**	Medialized (MGMH)						
*Edwards (2009)*		138	†	†	CTA (43%), revision (24%), MCT (7%)	7 (5.1%)	36
*Trappey (2011)*		284	108/176	†	†	17 (6%)	24
*Walch (2012)*		240	194/46	72	CTA (35%), MCT (14%), OA (11%)	7 (3.2%)	24
**Total**		662				31 (4.6%)	
**Lima SMR**							
*Bloch (2014)*	Medialized (MGMH)	133	41/92	69	CTA (79%), OA (11%), MCT (7%)	3 (2.2%)	38
**Exactech Equinoxe**							
*Friedman (2017)*	Minimally lateralized (MGLH)	591	†	69	MCT (25%), revision (24%), OA (18%)	3 (0.5%)	37
*Vourazeris*		202	†	71	†	3 (1.5%)	39
**Total**		793				6 (0.7%)	
**Multiple**							
*Benčić (2014)*	†	208	†	†	†	20 (9.6%)	12
*Glanzmann (2020)*		1480	460/1020	74	CTA (66%), MCT (9%), OA (6%)	8 (0.5%)	50
*Kang (2019)*		1649	†	64	CTA (39%), PHF (26%), MCT (8%)	9 (0.5%)	30
*Lehtimäki (2018)*		1904	241/1663	77	†	12 (0.6%)	32
*Merolla (2018)*		157	34/123	69	†	3 (1.9%)	49
*Russo (2015)*		195	66/129	67	†	1 (0.5%)	84
*Sebasan (2016)*		148	†	†	†	7 (4.7%)	30
*Wagner (2015)*		40	17/23	68	†	2 (5.0%)	37
*Wall (2007)*		240	†	75	CTA (30%), MCT (17%), OA (13%)	14 (7%)	24
**Total**		6021				77 (1.2%)	
**Unspecified**							
*Cheung (2018)*	†	119	47/72	71	CTA (55%), PHF (30%), revision (12%)	11 (9.2%)	27
*Dillon (2020)*		157	†	69	†	77 (49%)	30
**Total**		276				88 (31.8%)	

*MGMH*, medialized glenoid, medialized humerus; *MGLH*, medialized glenoid, lateralized humerus; *CTA*, Cuff tear arthropathy; *MCT*, massive cuff tear; *PHF*, proximal humerus fracture; *OA*, osteoarthritis.

∗ Top three most common diagnoses.

† Data not reported in original manuscript.

### Primary vs. revision RSA

Overall, in 7885 cases, there were 204 dislocations (2.6%). Specifically, there were 99 dislocations (35%) in 281 revision cases. The rate of instability reported ranged from 0.4 to 49%. Instability rates ranged from 1 to 5% (reporting only primary RSA cases), 1 to 49% (revision RSA cases only), and 0.4 to 10% (studies combining both).

### Implant type and design

Evaluating instability by implant type, six studies were identified that utilized one implant type,^3^^,^^13^^,^^14^^,^^33^^,^^36^ nine studies used multiple implant types,^2^^,^^18^^,^^19^^,^^21^^,^^23^^,^^26^^,^^27^^,^^35^^,^^37^ and two studies did not specify implant type (Table I).^8^^,^^11^ Instability rates by implant type ranged from 5.1 to 5.8% (Tornier Aequalis), 2.2% (Lima SMR), 0.5 to 1.5% (Exactech Equinoxe).^3^^,^^13^^,^^14^^,^^33^^,^^36^ Instability rates for studies utilizing multiple implants ranged from 0.5 to 9.6% and 9.2 to 49.0% for those unspecified. Two studies evaluated the effect of glenosphere size and design on dislocation rates.^8^^,^^27^ The rates of instability were 6.3% (5/80), 2.8% (2/52), 0% (0/16) for 40 mm, 36 mm, and 42 mm, respectively.^27^ Cheung et al. found no significant difference in mean (SD) glenosphere size in their instability cohort (38.5 mm, 2.8 mm) and stable cohort (37.1 mm, 2.3 mm), *P* = .06.^8^ Cheung et al. found a medialized glenosphere design to be a more stable implant with a dislocation rate of 2.1% (2/93) compared to 35% (9/26) in a lateralized design.^8^ In a multivariate regression model, they found 0.0036 lower risk (odds ratio) when using a medialized glenoid design comparatively.

### Subscapularis status

Subscapularis status as a risk factor for instability following reverse shoulder replacement was evaluated across all studies and five studies were found to have examined it specifically. Five studies compared instability rates in setting of subscapularis insufficiency or nonrepair versus successful subscapularis repair (Table II).^8^^,^^13^^,^^14^^,^^33^^,^^34^ Notably, the reason for no repair was due to a mix of surgeon choice or irreparable tendon in two studies.^14^^,^^34^ The range of instability rates reported was 1.2-19% with subscapularis deficiency and 0-1.7% with subscapularis repair.

**Table II:** Effect of subscapularis repair on instability rate.

Author, year	N, dislocated (N, total)	Instability Rate
*Subscapularis repaired*		
Cheung, 2018	1 (57)	1.7%
Edwards, 2009	0 (62)	0%
Friedman, 2017	0 (340)	0
Trappey, 2011	1 (161)	0.6%
Vourazeris, 2017	0 (86)	0%
***Total***	**2 (706)**	**0.2%**
Subscapularis insufficiency		
Cheung, 2018	10 (51)	19%
Edwards, 2009	7 (76)	9%
Friedman, 2017∗	3 (251)	1.2%
Trappey, 2011	14 (123)	12%
Vourazeris, 2017∗	3 (116)	2.6%
***Total***	**37 (617)**	**5.9%**

∗ Unrepaired subscapularis tendons were due to a mix of insufficiency and intentional decision to not repair.

### Proximal humerus fracture

Relating to preoperative diagnosis, three studies that analyzed diagnoses as risk factors for instability found proximal humerus fracture sequelae (nonunion, malunion) to be associated with a higher risk of instability 28-55%.^8^^,^^19^^,^^33^ Trappey et al. reported a significantly higher rate of instability in the fracture sequelae group than all other diagnoses (including acute fracture) combined (28% vs. 2%, *P* < .001).^33^ The collective instability rate among these three studies was 40% (16/40) in patients who underwent reverse shoulder arthroplasty for proximal humerus fracture nonunion or malunion.^33^

### Initial management

Management of instability after the index arthroplasty procedure was reported in eight studies (Table III). In three of the studies, closed reduction with component retention and thoracobrachial bracing or casting for at least six weeks was used as the primary management in 25 to 73% of primary arthroplasty cases.^13^^,^^18^^,^^33^ Achievement of a stable implant via closed reduction ranged from 28% to 100% in three studies.^13^^,^^18^^,^^33^ One of these studies also used this technique in 66% of their revision arthroplasty cases for postoperative instability with 25% success rate.^33^ Three studies utilized open reduction and brace or cast immobilization in 14-100% of primary cases in two studies and 17% of revision cases.^2^^,^^33^^,^^36^ Seven studies reported using revision arthroplasty with liner augmentation only in 27-100% of primary cases and 17% of revision cases.^3^^,^^8^^,^^13^^,^^18^^,^^33^^,^^35^^,^^36^ Stability was achieved in 55 to 100% of cases managed following revision RSA. Three studies achieved a stable prosthesis in 100% of cases after a single closed or open procedure.^2^^,^^13^^,^^35^

**Table III:** Management of instability cases.

Author (year)	N	Instability (N, %)	Timing of instability	Initial management (N,%)	Recurrent instability (N, %)	Subsequent management of recurrent instability (N)	Mean (range) no. Procedures	Final outcome – N (%) stable RSA
*Benčić (2014)*	208	20 (9.6%)	Early (< 4 weeks): 18 (90%) Late (>4 weeks): 2 (10%)	Open reduction and 4 weeks thoracobrachial casting (20, 100%)	0 (0%)	∗	∗	8 (100%)
*Bloch (2014)*	133	3 (2.2%)	Early (<1 week): 2 (66%) Late: (undefined): 1	Liner augmentation (2, 66%)	1 (33%)	Cuff-tear arthropathy reconstruction (1, 100%)	∗	3 (100%)
*Cheung (2018)*	119	11 (9.2%)	Mean (range) 8 weeks (3d-5mo)	Liner augmentation (11, 100%)	5 (45%)	Constrained liner (1, 20%) Revision RSA (1, 20%) Hemiarthroplasty (1, 20%) Resection arthroplasty (2, 40%)	2.8 (2-5)	8 (72%)
*Edwards (2009)*	138	7 (5.1%)	∗	Closed reduction + bracing x 6 weeks (2, 28%) Liner augmentation (5, 72%).	0 (0%)	∗	∗	7 (100%)
*Glanzmann (2020)*	1480	8 (0.5%)	∗	Closed reduction (3, 38%) Liner augmentation (5, 62%)	1 (12.5%)	Hemiarthroplasty (1, 100%)	2 (2)	7 (87.5%)
*Trappey (2011)*	Total (284) *Primary* (212) *Revision* (72)	17 (13%) 11 (5%) 6 (8%)	Median (range) 2 wk (1wk-3 yr) 1.5 wk (1wk-3yr) 2 wk (2-6 wk)	*Primary RSA* Closed reduction + bracing (8, 73%) Liner augmentation (3, 27%) *Revision RSA* Closed reduction + bracing (4,66%) Open reduction + bracing (1,17%) Liner augmentation (1,17%)	Overall 9 (53%) Primary RSA 5 (45%) Revision RSA 4 (66%)	*Primary* Closed reduction, brace (2, 40%) Revision RSA (3, 60%) *Revision* Revision RSA 1(4, 100%)		*Overall:* 9 (53%) *Primary:* 5 (45%) *Revision:* 4 (66%)
*Wagner (2015)*	Revision (40)	2 (5%)	∗	Liner augmentation (2, 100%)	0 (0%)	∗	∗	2 (100%)
*Walch (2012)*	240	7 (3.2%)	∗	Open reduction (1, 14%) Liner augmentation (4, 57%) Unspecified (2, 29%)	∗	∗	∗	7 (100%)

*RSA*, reverse shoulder arthroplasty; *d*, days; *wk*, weeks; *mo*, months; *yr*, years.

∗ Denotes missing data from manuscript.

### Management of recurrent instability

Recurrent instability (2 or more dislocations) was noted in 13 to 53% across 4 studies, with all cases requiring reintervention.^3^^,^^8^^,^^18^^,^^33^ After two or more dislocations in primary RSA cohorts, patients were treated with a second attempt at closed reduction and bracing (40%), 'cuff-tear arthropathy prosthesis' (further elaboration not provided by author) (100%), revision RSA (20-60%), conversion to hemiarthroplasty (20-100%), and resection arthroplasty (40%).^3^^,^^8^^,^^18^^,^^33^ For their revision RSA subcohort, Trappey et al. addressed recurrent instability with revision arthroplasty in 100% of cases.^33^ Following 2 or more dislocations, a final, stable implant was achieved in 53 to 100% of cases when the index procedure was a primary RSA and 66% in revision cases.^3^^,^^8^^,^^18^^,^^33^

## Discussion

This systematic review highlights that instability after RSA is highly variable based on multiple clinical factors discussed above. We found an overall instability rate of 2.5% in 7885 cases. In other recent systematic reviews, Ascione et al. found a 0.8% rate in modernized lateralized onlay humeral stems (0.8%), and Kennedy et al. reported ∼2% in all comers, reviews. Instability occurs infrequently following primary RSA but more commonly following revision RSA and management of proximal humerus fracture and fracture sequelae (nonunion and malunion). Subscapularis repair appears to be protective, aiding in stability of the prosthesis. For treatment of instability, both closed reduction with immobilization, as well as revision and liner augmentation, may result in a stable prosthesis. This review was the first to summarize the impact of numerous salient factors, including indication, subscapularis status, and implant design and size. Importantly, the review is the first to summarize salvage techniques for a persistently unstable RSA, showing high conversion rates to a stable prosthesis.

Kennedy et al. performed a systematic review of patient-reported outcomes and complications by preoperative diagnosis. In their study, they found that instability was highest in rheumatoid patients (5%), revision arthroplasty (1.8%), and proximal humerus fractures (1.7%).^20^ By diagnosis, Shah et al. found that failed arthroplasty (5.8%), proximal humerus fracture (4.1%), instability arthropathy (3.8%) had the highest rates of instability.^28^ They noted that revision RSA (5.7%) had higher rates than primary RSA (2.5%).^28^ In our systematic review, acute proximal humerus fractures and their sequelae (nonunion, malunion) demonstrated the highest rates of instability (28-55%) as compared primary RSA for all other indications (0.5-10%).^8^^,^^19^^,^^33^ Unfortunately due to limited data granularity in studies we reviewed, we could not stratify instability rates by other preoperative diagnoses. Numerous authors have commented on the root cause for instability after RSA for proximal humerus fractures. Tuberosity malunion, fracture nonunion, and bone loss following proximal humerus fracture may lead to soft tissue contracture and altered deltoid tensioning, subscapularis deficiency, and bony impingement, increasing the risk of instability following RSA.^8^^,^^33^ Attention intraoperatively to these risk factors may ameliorate the risk of instability, and further comparative studies are warranted.

Implant design plays an important role in the inherent stability of the prosthesis. Soft tissue tension through humeral distalization and/or relative glenosphere lateralization is thought to be paramount in increasing stability.^5^^,^^22^^,^^25^^,^^29^ In a recent systematic review, Shah et al. found that Grammont-style prostheses (medialized glenoid, medialized humerus) had significantly higher rates of instability (4%) than all others combined (1.3%), *P* < .001.^28^ In our review, only five studies evaluated one implant type, and nine studies that included multiple implants failed to present the data in such a way that allowed granular comparisons of instability rate by implant type or design. Using a descriptive analysis of implant design by Werthel et al. we were able to evaluate instability rates by implant design in the five studies that used one specific implant.^38^ A minimally lateralized design, the Exactech Equinoxe demonstrated the lowest instability rate (0.5%) in a series of 591 prostheses compared to the medialized designs, the Lima SMR (2.2%) in 133 prostheses, and Tornier Aequalis (5.1-6%) in 1462 prostheses.^3^^,^^13^^,^^14^^,^^33^^,^^36^ Cheung et al. did not specify implant type but noted that a medialized glenosphere design was more stable (2.1%, 2/93) compared to a lateralized design (35%, 9/26) in their series.^8^ Direct comparisons of different implant designs in larger studies deserves attention to better understand the role implant design plays in prostheses stability. However, based on the current pooled analyses available, it appears more contemporary lateralized designs decrease the risk of instability.

In our review of the literature, only two studies evaluated the effect of glenosphere size on the instability rate. Sabesan et al. reported rates of instability of 6.3% (5/80), 2.8% (2/52), 0% (0/16) for 40mm, 36mm, and 42 mm glenospheres, respectively. Cheung et al. found no significant difference in glenosphere size between dislocators and nondislocators.^8^ It is has been shown in biomechanical studies that a larger glenosphere size improves impingement free range of motion and theoretically increases inherent stability; still, others believe glenosphere size is secondarily important in providing stability compared to soft tissue tensioning and prosthesis constraint (i.e. glenosphere diameter to humeral socket radius ratio).^22^^,^^25^^,^^29^ Moreover, little attention has been paid to directly evaluating this factor on instability rates in large series of reverse shoulder arthroplasties and remains a variable of interest in the future study of instability.

Whether to repair or augment the subscapularis tendon during RSA remains a topic of controversy. A prior biomechanical study suggests a significant benefit in repairing the subscapularis tendon, resulting in higher force required to dislocate anteriorly.^24^ However, certain lateralized implant designs may make subscapularis repair difficult and subsequent subscapularis deficiency inevitable.^38^ Clark et al. reviewed their experience and found no difference in dislocation rate between patients with subscapularis repair or nonrepair (3.2 vs. 5.7%, *P* = .52).^9^ Chalmers et al. found a high rate of subscapularis deficiency (64%) in a subgroup of RSA patients with instability.^7^ In this systematic review, five studies evaluated instability following subscapularis repair versus deficiency. Subscapularis deficient cases overall had a higher mean (5.9%) and range (1.2-19%) rates of instability compared to subscapularis repair (0.2%, 0-1.7%). The clinical equipoise calls into question the utility and necessity of tendon transfer or allograft reconstruction when the subscapularis is deficient or irreparable.

Revision surgery for reverse shoulder arthroplasty often comes with a higher risk of complications due to scar tissue and soft tissue contracture, proximal humeral bone loss, and heterotopic bone.^6^ The risk of instability following RSA is cited to be as high as 36% according to a large multicenter study evaluating the epidemiology and etiology of failed shoulder arthroplasty by Gauci et al.^16^ In this systematic review of 7885 cases there were 281 revisions (3.5%). There were 99 cases of instability, accounting for a 35% instability rate following revision surgery, consistent with instability rates of the previous study.

Instability following RSA remains a challenging clinical problem with few viable salvage options. Recurrent instability requiring reintervention has been cited as high as 26 to 29%, and overall complications following revision surgery as high as 50%.^16^^,^^31^^,^^32^ Despite the breadth of literature on reverse shoulder arthroplasty, the initial management of instability remains a topic of clinical debate. The efficacy of closed reduction and immobilization has been evaluated in previous studies. Chalmers et al. reported a 44% (4/9 of cases) success rate following closed reduction and immobilization in an abduction orthosis.^7^ Teusink et al. reported 62% (13/21 cases) success with closed reduction and no difference in success between early or late dislocators. All remained stable at 28 months follow-up.^32^ They concluded that all early dislocators should undergo attempted closed reduction as the long-term ASES scores did not significantly differ from those patients treated with revision surgery. In this review, 0-70% of cases were initially managed with closed reduction and immobilization with success varying from 28 to 100%.^13^^,^^18^^,^^33^ Gerber argued that early dislocation (<90 days postsurgery) was due to technical error and revision surgery is advised to address component malposition or inadequate soft tissue tensioning. Still, Boileau recommends an attempt at closed management for early dislocations in spite of recognizing limited efficacy in their own series (59%).^4^^,^^17^

In the setting of failed closed reduction, suspected component malpositioning, or inadequate soft tissue tensioning, open reduction and revision surgery are indicated. Gallo et al. reported on nine cases of RSA instability in which all were treated with revision surgery, 4 patients undergoing multiple revisions. At final follow-up, 66% (6/9) of RSAs were explanted or chronically dislocated at final follow-up.^15^ Chalmers reported on their patients with persistent instability after closed reduction treated with revision to a thicker polyethylene insert, and found 82% (9/11) stable constructs at final follow-up.^7^ Teusink et al. also reported on a cohort of 8 patients who failed closed reduction, with 75% (6/8) achieving stable constructs with humeral and glenosphere component exchange and 25% (2/8) of prosthetics persistently unstable.^32^ In four studies reporting recurrent instability requiring reintervention, revision of the humeral or glenosphere components was performed in 20-60% while other common interventions were hemiarthroplasty (20-100%) and resection arthroplasty (0-40%).^3^^,^^8^^,^^18^^,^^33^

**Figure 2:**
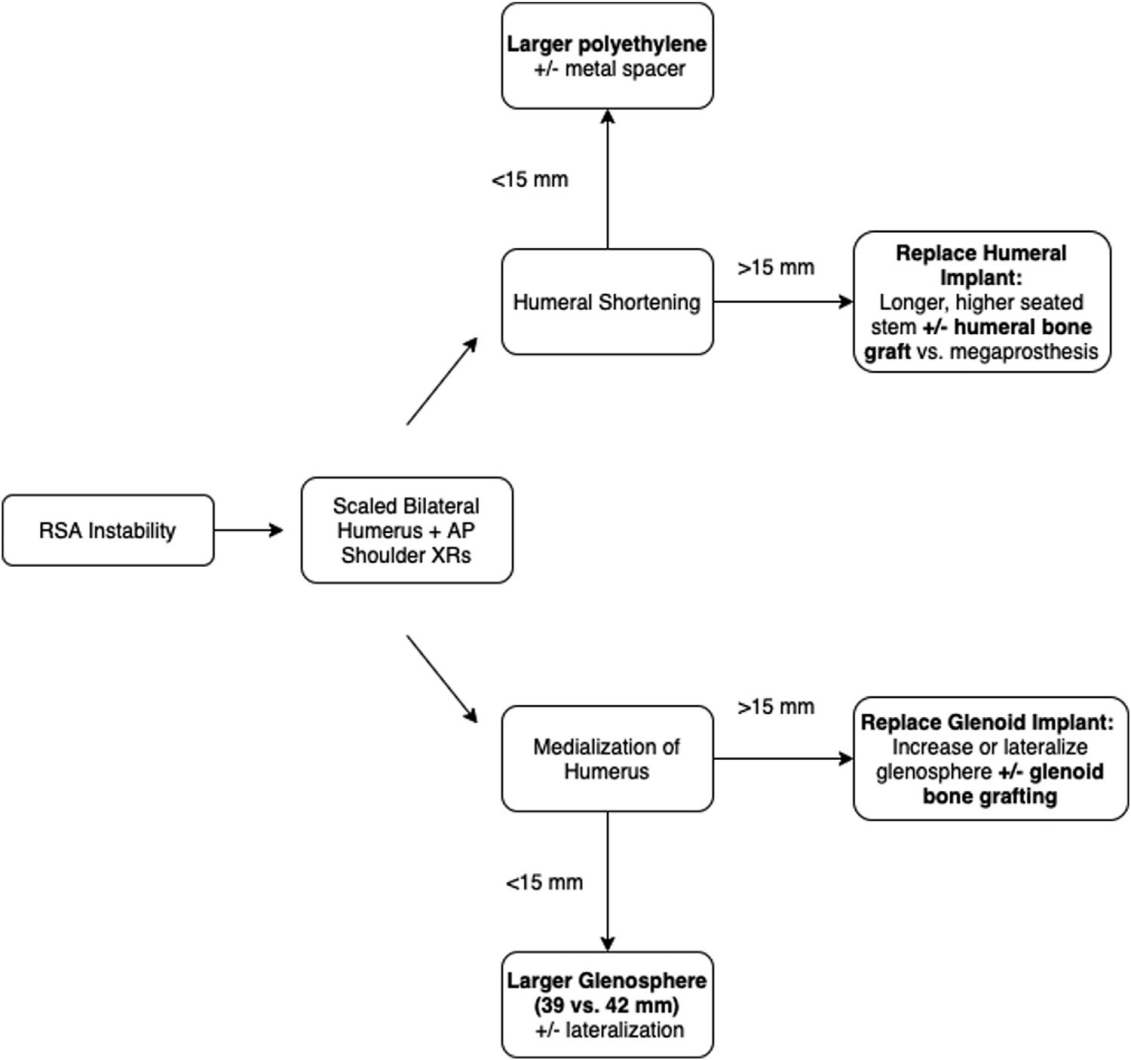
Management Algorithm for an Unstable Reverse Shoulder Arthroplasty. *RSA,* reverse shoulder arthroplasty. Figure adopted from P. Boileau, Orthopaedics & Traumatology: Surgery & Research (2016).

Understanding the most likely etiology of instability to appropriately address the issue is critically important during revision surgery to achieve a stable implant. Inadequate deltoid tensioning in the vertical (humeral shortening) or horizontal plane (excess medialization) is the usual etiology of instability; however, component malposition (humeral version or baseplate position) should not be overlooked.^4^ Boileau proposes a treatment algorithm for instability, which we present for illustration of how to manage instability based on the underlying etiology (Fig. 2).^4^ Metal spacers and thicker polyethylene liners, longer, higher humeral stems, and humeral bone grafting may restore humeral length based on the amount of initial shortening. Larger glenospheres and glenoid augments increase deltoid wrapping through lateralization, improving compressive forces and thus prosthesis stability. Ultimately, we recommend an attempted closed reduction and period of immobilization. However, in the setting of persistent dislocation and mechanical concerns, the surgeon should think critically about the etiology and address the underlying issue during the revision surgery.

## Limitations

There are limitations of this study secondary to its design and the articles that were reviewed. The majority of studies were level III or IV evidence studies, and further, by the MINORS criteria, the quality of evidence was calculated to be of low to fair quality, limiting the overall level of strength of this systematic review. The authors attempted to summarize all causes for instability after RSA, including primary and revision cases. As such, the cohort is heterogeneous with respect to preoperative indication for surgery, demographics, and implant types and designs. This inherently limits the ability to perform a large-scale metanalysis with the data available. Further, in some studies, there was no mention of salient factors that may contribute to instability. For example, in many studies, there were multiple implants used but no stratification of instability risk by implant type despite having fundamental differences in design. While these limitations exist, this study represents an analysis of the largest cohort of pooled cases of instability after RSA. Lastly, we cannot rule out the effect of advances in technology and improved prostheses, which may impact rates of instability. In addition, in the era of highly subspecialized training, these rates may not be generalizable, and the true underestimate of the true rate of instability may be much higher in the hands of generalist orthopedic surgeons.

## Conclusion

In this systematic review, instability infrequently occurred in primary RSAs (1-5%), and commonly in revision RSAs (1-49%). Indication of acute fracture, as well as fracture sequelae, has a high rate of instability. While successful management of RSA instability can be achieved with closed versus open reduction and immobilization or component revision, some patients may ultimately require hemiarthroplasty or resection arthroplasty.

## Disclaimers:

Funding: No funding was disclosed by the authors.

Conflicts of interest: The authors, their immediate families, and any research foundation with which they are affiliated have not received any financial payments or other benefits from any commercial entity related to the subject of this article.
